# Editorial Note: Analysis of the high-quality development path of China’s tea export

**DOI:** 10.1371/journal.pone.0342827

**Published:** 2026-02-17

**Authors:** The PLOS One Editors

The *PLOS One* Editors issue this Editorial Note because this article [1] was identified as one of a series of submissions for which we have concerns about aspects of the peer review process. Readers are advised to interpret the article [1] with caution.

The Editors have no evidence of any author involvement in the peer review concerns.

## References

[1] Qin K, Zhou L. Analysis of the high-quality development path of China’s tea export. PLoS One. 2024;19(11):e0311629. doi: 10.1371/journal.pone.0311629 39531420 PMC11556720

